# *Libyan Journal of Medicine* among top journals in African and Arab countries

**DOI:** 10.3402/ljm.v8i0.20686

**Published:** 2013-03-26

**Authors:** Omran Bakoush, Amin Bredan, Hani Benamer, Mohamed Daw

**Affiliations:** 1 Department of Internal Medicine, UAE University, UAE & Department of Nephrology, Lund University, Sweden; 2 Ghent University, Ghent, Belgium; 3 Neurology Department, New Cross Hospital, Wolverhampton WV10 OQP, United Kingdom; 4 University of Tripoli, Tripoli, Libya

African and Arab academic and research institutions contribute modestly to the international biomedical literature. Inadequate research funding and limited national, regional and international collaboration are important factors contributing to this deficiency. Furthermore, only few regional journals are covered by major indexing systems such as MEDLINE (Index Medicus), which hampers the international dissemination of research results obtained in African and Arab nations.

To increase the international visibility of regional research data, *Libyan Journal of Medicine* (LJM) was established in 2006 as an open access journal. Since then, its scientific status has steadily improved. In addition to the less selective databases, it is currently indexed in the most prestigious citations databases: Embase, Scopus, Thomson's Science Citation Index, PubMed Central, and MEDLINE. In a relatively short time, it has gained a promising record of citations compared to its peers (Table 1). LJM has a 2-year impact factor of 0.29 and a Google scholar H5-index for the articles published in the last 5 years of 9. Both of these sit at the median of the selected 31 general medicine journals from Arab and African nations listed in Scopus (Table 1). Moreover, a substantial number of its research articles are characterized by international collaborations, ranking third among the listed journals.

**Table 1:** The date of the first publication and the bibliometric data of the 31 general medicine journals from Arab and African nations listed in Scopus database

Journal	First published	H5-index^1^	Cites/doc^2^ (2 years)	% IC^3^
Journal of the Egyptian Public Health Association	1926	5	0.33	0.0
Tunisie Medicale	1929	8	0.28	1.5
South African Medical Journal	1932	24	1.23	16.0
The Lebanese Medical Journal	1950	8	0.32	8.3
Ethiopian Medical Journal	1962	9	0.59	19.1
Le Mali Médical	1963	2	0.03	0.0
Jordan Medical Journal	1965	2	0.07	0.0
African Journal of Medicine and Medical Sciences	1976	8	0.38	0.0
Saudi Medical Journal	1979	20	0.56	8.5
Bahrain Medical Bulletin	1979	NA	0.12	4.5
Qatar Medical Journal	1980	NA	0.00	10.0
West African Journal of Medicine	1981	8	0.35	7.7
Annals of Saudi Medicine	1985	17	1.24	7.6
Medical Principles and Practice	1988	19	1.04	12.7
Journal of the Bahrain Medical Society	1989	NA	0.02	0.0
Malawi Medical Journal	1990	6	0.29	18.5
Nigerian Quarterly Journal of Hospital Medicine	1990	8	0.27	0.0
Nigerian Journal of Medicine	1990	10	0.38	0.0
South African Family Practice	1994	10	0.19	10.0
Nigerian Postgraduate Medical Journal	1994	8	0.24	0.0
Eastern Mediterranean Health Journal	1995	17	0.56	25.3
Nigerian Journal of Clinical Practice	1998	9	0.29	4.9
African Journal Biomedical Research	1998	NA	0.12	7.7
Kuwait Medical Journal	1998	NA	0.05	13.3
Sultan Qaboos University Medical Journal	1999	5	NA	14.0
African Health Sciences	2001	14	0.58	27.3
Annals of African Medicine	2001	10	0.84	6.0
East African Journal of Public Health	2004	11	0.24	0.0
New Iraqi Journal of Medicine	2005	NA	0.03	10.2
Libyan Journal of Medicine	2006	9	0.29	25.0
International Journal of Health Research	2008	NA	0.46	7.7

Data are from Scimago Journal and Country Rank (http://www.scimagojr.com/ retrieved February 9, 2013), Journals Catalog of the National Library of Medicine, and Google Scholar.

1 H-index for articles published in the last 5 years.

2 Average number of citations per document in a 2-year period.

3 Percent international collaboration (documents with authors from more than one country address).

In 2012, the journal received 250 manuscript submissions, of which only the scientifically most qualified were published: only 15% of the submitted papers were selected for publication. We are thankful to the enormous services provided by our editors, reviewers, and publisher, whose cooperation has enabled LJM to become a well-established and respected channel for publishing research of both regional and international interest from various parts of the world. LJM will continue its policy of publishing only high-quality research that makes significant contributions to the body of knowledge. The editors are keen on attracting even more high-quality papers from regional research groups. To that end, raising the efficiency of the peer review process, expanding the editorial board, and broadening the editors’ collective expertise are essential steps. We have already embarked on that road.

*Omran Bakoush* Department of Internal Medicine UAE University, UAE & Department of Nephrology Lund University, Sweden Email: omran.bakoush@med.lu.se*Amin Bredan* Ghent University, Ghent, Belgium*Hani Benamer* Neurology Department, New Cross Hospital Wolverhampton WV10 OQP, United Kingdom*Mohamed Daw* University of Tripoli, Tripoli, Libya

